# Supplementary material for: Molecular dynamics simulations of the calmodulin-induced α-helix in the SK2 calcium-gated potassium ion channel
Source: J Biol Chem. 2022 Dec 29;299(2):102850. doi: 10.1016/j.jbc.2022.102850 (PMC9874072; doi:10.1016/j.jbc.2022.102850)
Supplement: Supporting Figures S1–S7 and Table S1 [file mmc1.docx]

**Molecular dynamics simulations of the calmodulin-induced α-helix in the SK2 calcium-gated potassium ion channel**

**Rafael Ramis,^*,1,2^ Óscar R. Ballesteros,^2,3^ Arantza Muguruza-Montero,^4^ Sara M-Alicante,^2,4^ Eider Núñez,^2,4^ Álvaro Villarroel,^4^ Aritz Leonardo,^1,2^ and Aitor Bergara^1,2,3^**

^1^Donostia International Physics Center, 20018 Donostia, Spain

^2^Departamento de Física, Universidad del País Vasco, UPV/EHU, 48940 Leioa, Spain

^3^Centro de Física de Materiales CFM, CSIC-UPV/EHU, 20018 Donostia, Spain

^4^Instituto Biofisika, CSIC-UPV/EHU, 48940 Leioa, Spain

^*^E-mail: rafael.ramis@dipc.org

**Supporting Information**

**Contents**

**Figure S1**: metadynamics results.

**Figure S2**: assessment of the convergence of metadynamics.

**Figure S3**: sequence alignment of different CaMBDs to the hA of the SK channel family.

**Figures S4 and S5**: secondary structure analysis of the other SK family members and other CaMBDs showing IQ motifs.

**Figure S6**: Sequence of the SK2 CaMBD, colored according to the conservation score of each residue.

**Figure S7**: Trajectories of the different Hamiltonian replica exchange replicas in the effective temperature space.

**Table S1**: Evolutionary rates for all residues in the SK2 CaMBD ordered core region.


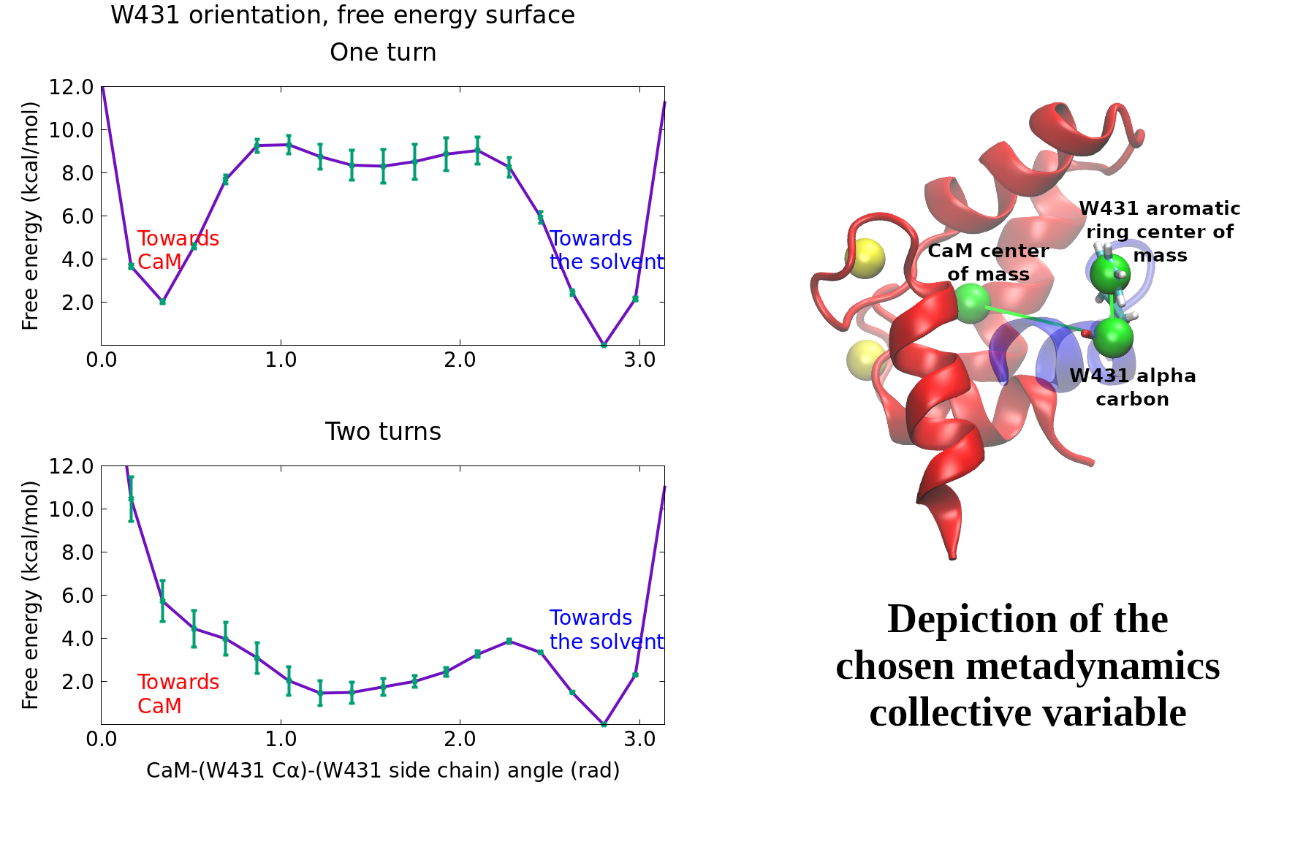


**Figure S1**: Metadynamics results of the “one turn” (top) and the “two turns” (bottom) systems. Free energy surfaces as a function of the angle between the C-lobe of CaM, the C_α_ of W431, and its aromatic ring (the angle determined by the green spheres on the right panel), along a 2500-ns simulation for each system. The surfaces are the average over the last 250 ns of simulation, and the error bars represent their standard deviation.


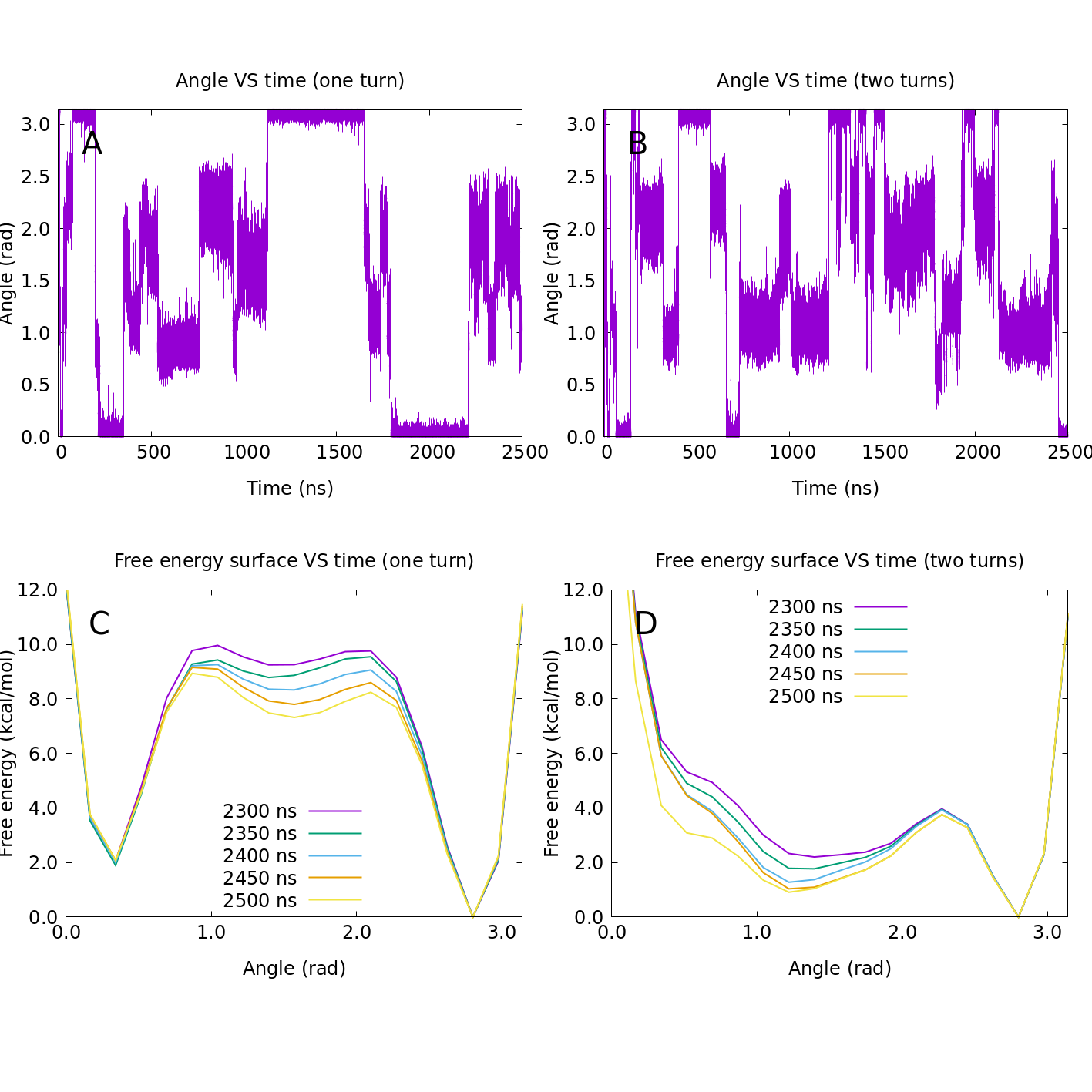


**Figure S2**: Metadynamics convergence. A and B: Time variation of the collective variable (angle between the C-lobe of CaM, the C_α_ of W431 and its aromatic ring) along a 2500-ns simulation for the “one turn” and the “two turns” systems, respectively. C and D: Potential free energy surfaces projected on this collective variable along the last 250 ns of simulation for the “one turn” and the “two turns” systems, respectively. These surfaces were used to compute the final barrier.


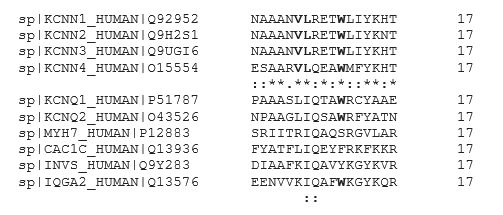


**Figure S3**: Sequence alignment of different CaMBDs to the hA of the SK channel family, carried out with the Clustal Omega tool (1).


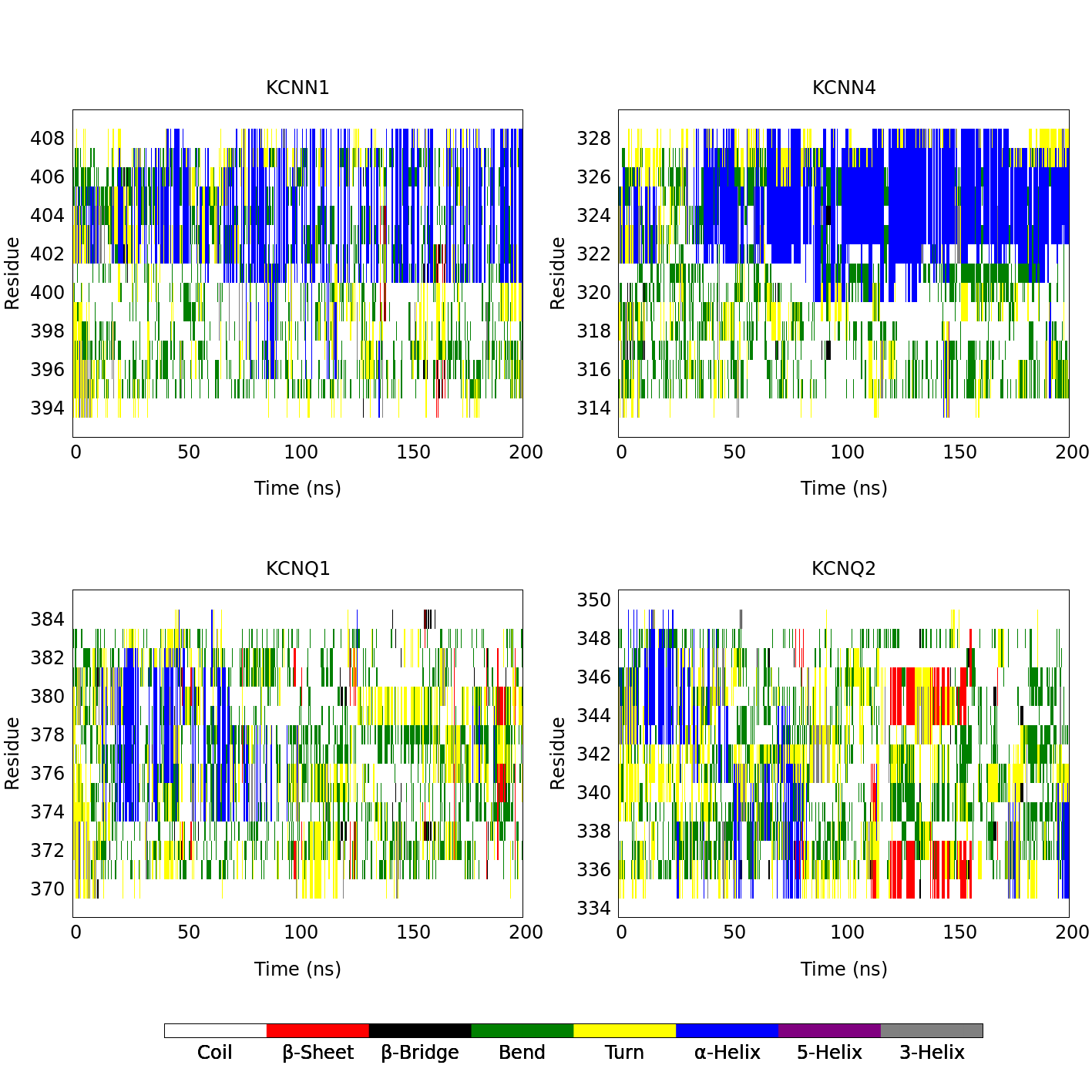


**Figure S4**: Secondary structure content of the other SK channels (KCNN1 (SK1) and KCNN4 (SK4)), as well as the related KCNQ1 and KCNQ2, in aqueous solution in the absence of CaM, calculated as in our previous work (2).


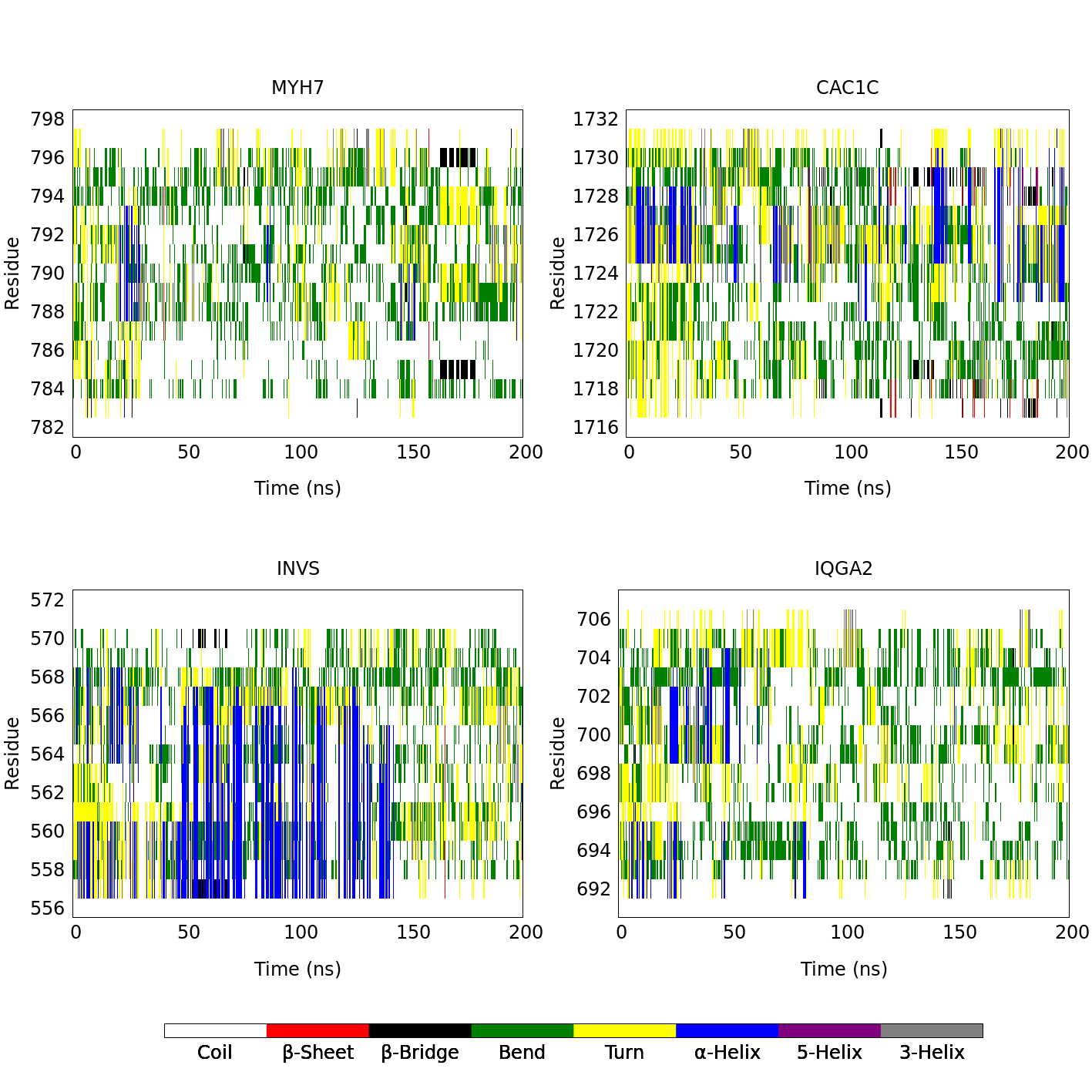


**Figure S5**: Secondary structure content of some additional CaM targets showing IQ motifs: MYH7, CAC1C, INVS, and IQGA2, in aqueous solution in the absence of CaM, calculated as in our previous work (2).


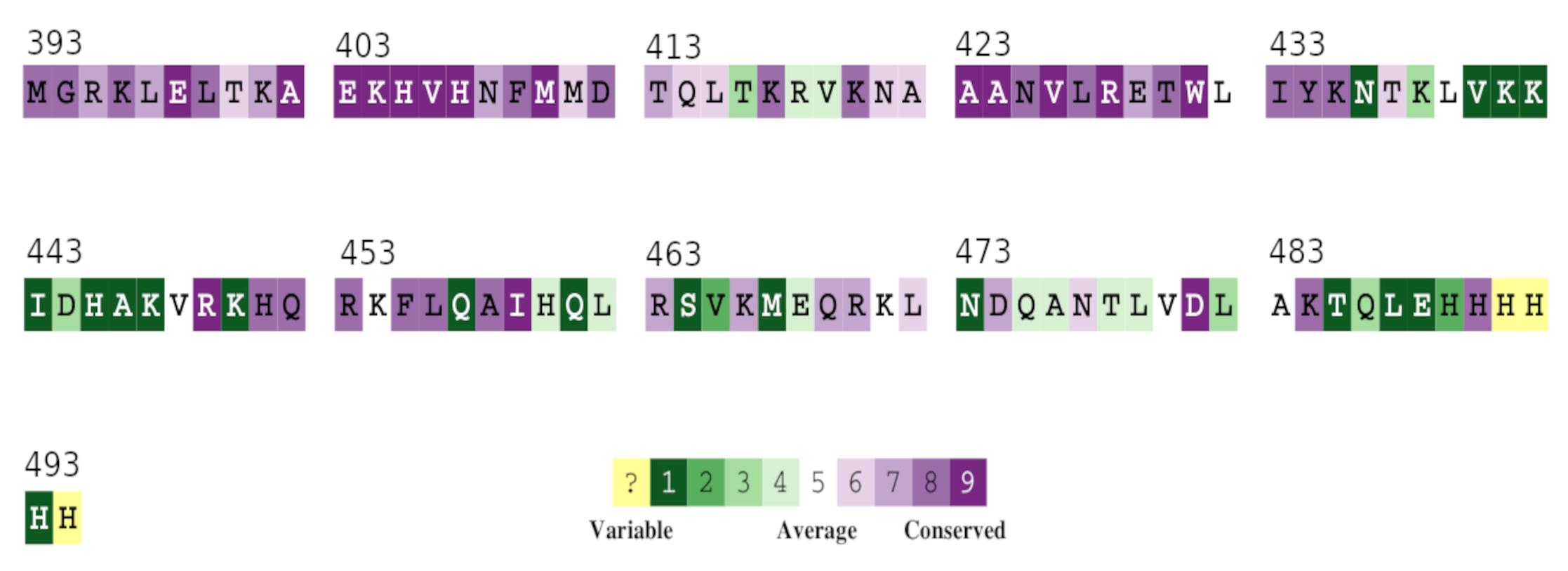


**Figure S6**: Sequence of the SK2 CaMBD, colored according to the conservation score of each residue, calculated using the ConSurf server (<https://consurf.tau.ac.il/consurf_index.php>) (3–7) with default parameters. The results were already pre-calculated and available in the ConSurf-DB database (<https://consurfdb.tau.ac.il/main_output.php?pdb_ID=1KKD&view_chain=A&unique_chain=4J9YB&cbs=1>) (8, 9). The simulated region spans the N421-T437 segment. Residues with the highest conservation scores (in purple) are those with the slowest evolutionary rates (i.e. the most conserved), whereas those with the lowest (in green) are those with the fastest (i.e. the most variable). Residues with unreliable scores due to a high uncertainty are colored in yellow.


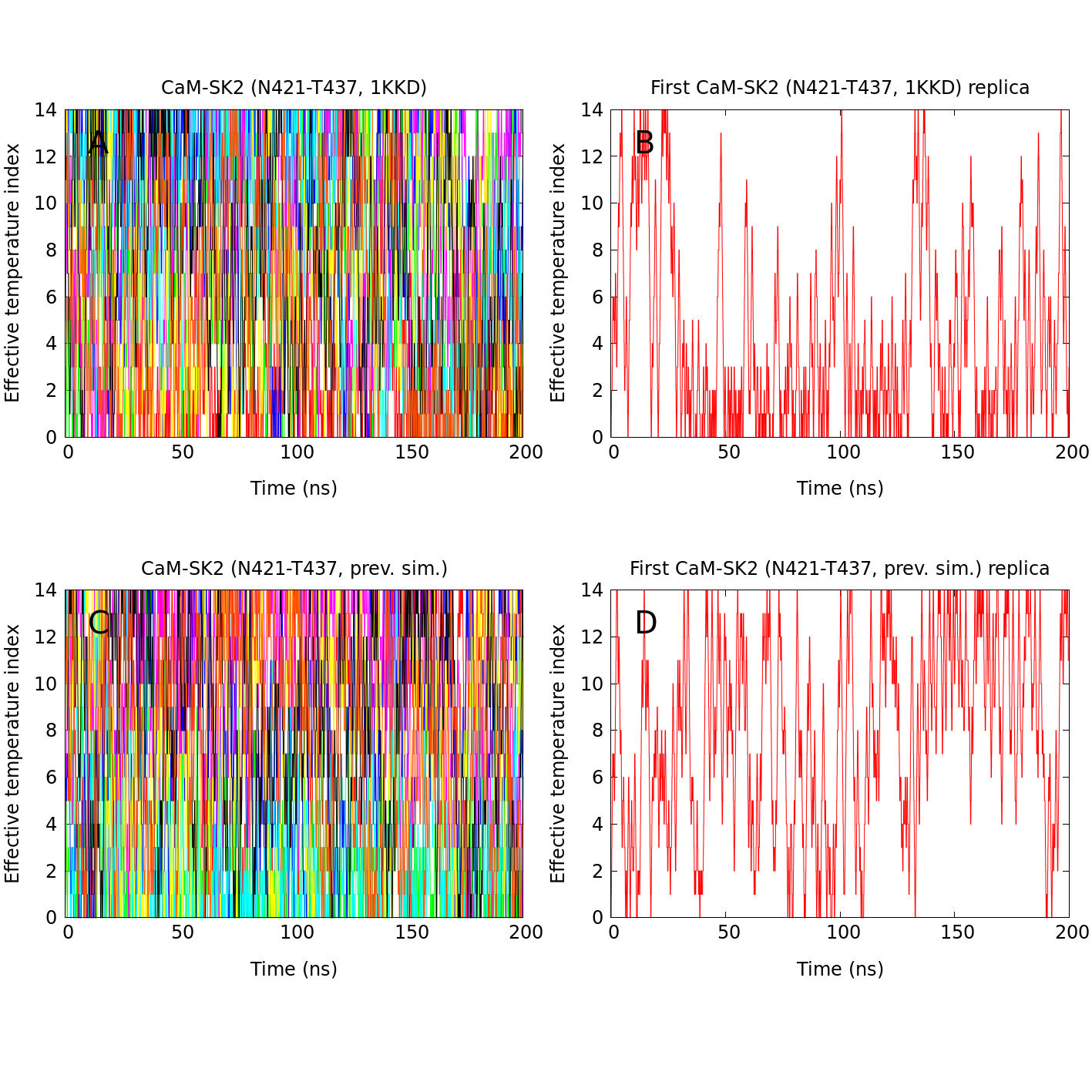


**Figure S7**: Trajectories of all replicas (A and C) and of just the first replica (B and D) in the effective temperature space for the simulations starting from the 1KKD SK2 coordinates (A and B) and from one of the final snapshots in our previous SK2 simulations in the absence of CaM (2) (C and D).

**Table S1**: 50% confidence intervals for the normalized evolutionary rates for all residues in the ordered core region of the SK2 CaMBD (the N421-T437 fragment), relative to the whole CaMBD, as calculated using the ConSurf server (<https://consurf.tau.ac.il/consurf_index.php>) (3–7) with default parameters.

| **Residue** | **Evolutionary rate 50% confidence interval** |
| --- | --- |
| N421 | (-0.397, -0.043) |
| A422 | (-0.322, -0.043) |
| A423 | (-1.135, -1.078) |
| A424 | (-1.135, -1.078) |
| N425 | (-0.827, -0.643) |
| V426 | (-1.078, -0.969) |
| L427 | (-0.969, -0.786) |
| R428 | (-1.028, -0.903) |
| E429 | (-0.741, -0.530) |
| T430 | (-0.937, -0.786) |
| W431 | (-1.135, -1.028) |
| L432 | (-0.238, -0.208) |
| I433 | (-0.827, -0.643) |
| Y434 | (-0.903, -0.694) |
| K435 | (-0.903, -0.741) |
| N436 | (0.801, 1.643) |
| T437 | (-0.466, -0.147) |

**References**

1. Madeira, F., Park, Y. M., Lee, J., Buso, N., Gur, T., Madhusoodanan, N., Basuktar, P., Tivey, A. R. N., Potter, S. C., Finn, R. D., and Lopez, R. (2019) The EMBL-EBI search and sequence analysis tools APIs in 2019. *Nucleic Acids Res.* **47**, W636–W641

2. Muguruza-Montero, A., Ramis, R., Núñez, E., Ballesteros, O. R., Ibarluzea, M. G., Araujo, A., M-Alicante, S., Urrutia, J., Leonardo, A., Bergara, A., and Villarroel, A. (2021) Do calmodulin binding IQ motifs have built-in capping domains? *Protein Sci.* **30**, 2029–2041

3. Ashkenazy, H., Abadi, S., Martz, E., Chay, O., Mayrose, I., Pupko, T., and Ben-Tal, N. (2016) ConSurf2016: an improved methodology to estimate and visualize evolutionary conservation in macromolecules. *Nucleic Acids Res.* **44**, W344–W350

4. Celniker, G., Nimrod, G., Ashkenazy, H., Glaser, F., Martz, E., Mayrose, I., Pupko, T., and Ben-Tal, N. (2013) ConSurf: using evolutionary data to raise testable hypotheses about protein function. *Isr. J. Chem.* **53**, 199–206

5. Ashkenazy, H., Erez, E., Martz, E., Pupko, T., and Ben-Tal, N. (2010) ConSurf2010: calculating evolutionary convservation in sequence and structure of proteins and nucleic acids. *Nucleic Acids Res.* **38**, W529–W533

6. Landau, M., Mayrose, I., Rosenberg, Y., Glaser, F., Martz, E., Pupko, T., and Ben-Tal, N. (2005) ConSurf2005: the projection of evolutionary conservation scores of residues on protein structures. *Nucleic Acids Res.* **33**, W299–W302

7. Glaser, F., Pupko, T., Paz, I., Bell, R. E., Bechor-Shental, D., Martz, E., and Ben-Tal, N. (2003) ConSurf: identification of functional regions in proteins by surface-mapping of phylogenetic information. *Bioinformatics* **19**, 163–164

8. Ben Chorin, A., Masrati, G., Kessel, A., Narunsky, A., Sprinzak, J., Lahav, S., Ashkenazy, H., and Ben-Tal, N. (2020) ConSurf-DB: an accessible repository for the evolutionary conservation patterns of the majority of PDB proteins. *Protein Sci.* **29**, 258–267

9. Goldenberg, O., Erez, E., Nimrod, G., and Ben-Tal, N. (2009) The ConSurf-DB: pre-calculated evolutionary conservation profiles of protein structures. *Nucleic Acids Res.* **37**, D323–D327
